# Spatial monitoring of hydrolysis in a plug-flow bioreactor: a support for flexible operation?

**DOI:** 10.1186/s40643-024-00740-0

**Published:** 2024-02-14

**Authors:** Theresa Menzel, Peter Neubauer, Stefan Junne

**Affiliations:** 1 Chair of Bioprocess Engineering, Institute of Biotechnology, Technische Universität Berlin, Ackerstraße 76, ACK 24, 13355 Berlin, Germany; 2 Department of Chemistry and Bioscience, Aalborg University Esbjerg, Niels Bohrs Vej 8, 6700 Esbjerg, Denmark

**Keywords:** Multiposition monitoring, Anaerobic digestion, Bedding straw, Thin-sludge recirculation, Hydraulic retention time

## Abstract

Hydrolysis at changing hydraulic retention time, recirculation, bedding straw content in the feed, bioaugmentation and the impact of those changes on gradient formation in the liquid phase in plug-flow reactors (PFRs) was examined. The pH-value, conductivity and oxidation–reduction potential (ORP) were monitored at three spots along the PFRs to study potential correlations to process performance during a total process time of 123 weeks. The *on-line* monitoring showed good correlations to acidogenesis: namely, the pH and ORP to the acidification, to butyric (and lactic) acid concentration and to the acid yield. The ORP (measured at the inlet) showed the most stable correlation to acidogenesis under dynamic operation, while the conductivity (at the outlet) correlated to the acid concentration in dependence on the feedstock. Multiple measurement spots as used in this study allow to gain more information about acidogenic fermentation than a single spot, simplifying process control and automation attempts with recalcitrant feedstock.

**Graphical Abstract:**

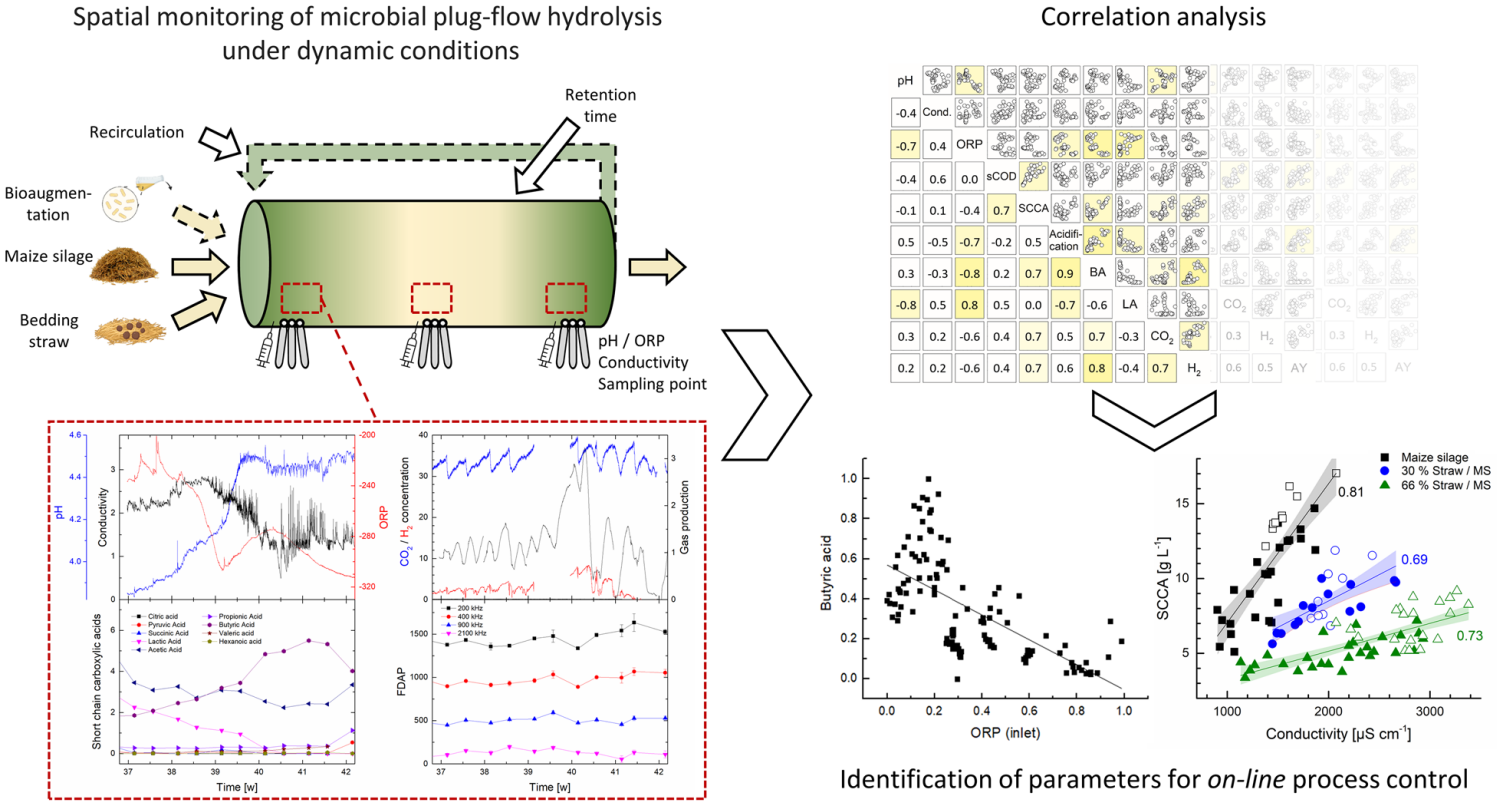

**Supplementary Information:**

The online version contains supplementary material available at 10.1186/s40643-024-00740-0.

## Introduction

Anaerobic digestion (AD) in two stages, in which the hydrolytic and acidogenic phase is spatially separated from methanogenesis, is of interest for improving the digestion of residual biomass. The two-stage setup holds various advantages depending on the feedstock: (i) a fast acidification of the methanogenesis is prevented in case of a sudden increase of easily degradable matter (e.g., food waste), (ii) the detoxification of inhibiting biomass such as citrus waste (Lukitawesa et al. 2018) or inhibitors from thermal pretreatment (Li et al. 2017); (iii) increased hydrolysis of recalcitrant biomass in the first stage under certain process conditions (e.g., acidic or alkaline pH-value) other than optimal for full digestion (Voelklein et al. 2016; Cheah et al. 2019), (iv) thermophilic pre-digestion (Garcia-Aguirre et al. 2017; Qin et al. 2017), respectively. Two-stage digestion thereby leads to higher yields, improved stability, and the possibility to generate multiple products like short-chain carboxylic acids (SCCAs) and hydrogen beside biogas, as summarized by Janesch et al. (2021). Since the hydrolytic and acidogenic microbial community is more robust towards changes in the environmental conditions, introducing a hydrolysis stage also offers a higher flexibility of the AD system, especially for the on-demand production of methane (Hahn et al. 2014; Linke et al. 2015). In large scale, feedstock availability and quality may change over time and thereby affect biogas production. In particular, two-stage digestion has been shown to deal better with shock-loadings and a fluctuating feed (Grimberg et al. 2015; Chatterjee and Mazumder 2019). The rapidly growing hydrolytic organisms also have a higher capability to adapt to changes in the feedstock’s composition (Van et al. 2020). So far, however, upscaling of two-stage digestion is often limited to the application of food waste (Menzel et al. 2020).

As AD is performed by mixed microbial communities, its kinetics can be unstable, especially if external changes of the feedstock or process conditions occur (Cruz et al. 2021). To ensure a stable AD system in large scale and with dynamic feeding of various feedstock, a suitable process monitoring strategy is required. Therefore, there is a rising interest in the *on-line* monitoring of AD processes, as it allows the early detection of process disturbances, and thus ensures a stable production of biogas although some relevant cultivation conditions may vary.

Until now, real-time monitoring in AD is limited to a very few parameters. Important signal parameters, such as the SCCA concentration, are usually not measured due to a lack of generally applicable strategies (Wu et al. 2019). While many precise and sensitive monitoring techniques exist, these are often too expensive for the large scale (spectroscopic techniques) or offer limited information (titration methods). In contrast, electrochemical sensors are a fast, convenient, and cheap method of monitoring, that has already been implemented frequently in pilot scale (Wu et al. 2019; Cruz et al. 2021). In lab-scale AD, correlations of the conductivity to the SCCA concentration (Aceves-Lara et al. 2012; Marín-Peña et al. 2020) and a linear dependency of the oxidation reduction potential (ORP) on the soluble chemical oxygen demand (sCOD) release (Chang et al. 2002) have been found—making these measurements a promising option for the determination of the hydrolytic and acidogenic efficiency.

In plug-flow reactors (PFRs) with laminar flow, mixing occurs in radial direction more or less exclusively. As a result, if there is sufficient hydrolytic and acidogenic activity among the microbial population, gradients should appear among the pH-value, ORP and conductivity. This was observed recently during the acidification and subsequent dark fermentation of maize silage (MS) and grass silage in a lab-scale PFR. Depending on the operation mode and position of the sensors along the PFR, correlations of the pH-value, conductivity and the ORP to the SCCA and gas concentration as well as to cell viability measures were found (Longis et al. 2023).

The formation of distinct process phases, in particular an acidogenic and methanogenic phase in PFRs, has been observed with various feedstock before (Sans et al. 1995; Namsree et al. 2012; Dong et al. 2019; Veluchamy et al. 2019; Khalil et al. 2021). However, the application of a PFR as separate hydrolysis stage under the formation of a distinct hydrolytic and acidogenic phase has not been investigated thoroughly yet. Prior to the current study, the authors reported about the digestion of MS mixed with bedding straw in such a process concept (Menzel et al. 2023a; b). Although hydrolysis was partly limited by a low pH-value, the formation of gradients and microenvironments due to a laminar flow in the PFR provided obviously suitable growth conditions for the microbial community. In the current study, the importance of spatial monitoring inside these PFRs during the anaerobic hydrolysis of lignocellulosic biomass was further investigated under variations of the feedstock composition, process conditions [hydraulic retention time (HRT) and thin-sludge recirculation] and with bioaugmentation of hydrolytic microorganisms, as described elsewhere (Menzel et al. 2023a; b). *On-line* monitoring of the pH-value, conductivity, and the ORP was applied in three distinct spots along the reactor length in a similar hardware setup as described recently when MS and gras silage was applied as feedstock (Longis et al. 2023).

The aim of this study was the investigation of gradient formation and the identification of suitable monitoring spots for individual parameters during the long-term dynamic operation of a hydrolytic PFR. During the whole operation period, measurements of the main metabolites of hydrolysis (sCOD) and acidogenesis (SCCA) were conducted. Furthermore, the ratio of acids within the sCOD (acidification), dominant products (butyric/lactic acid) as well as the specific yields and rates of hydrolysis and acidogenesis, along with the physiological state of the culture measured by frequency-dispersed anisotropy polarizability (FDAP), were determined. Spatial monitoring was further examined for its relation to process performance. Finally, results were summarized for the identification of the most suitable monitoring strategies to be deployed under dynamic conditions. The application of different modes of process operation and periods of dynamic operation should ensure a wide process variability, and thus applicability, of our findings.

## Materials and methods

### Spatial on-line monitoring

**Fig. 1:**
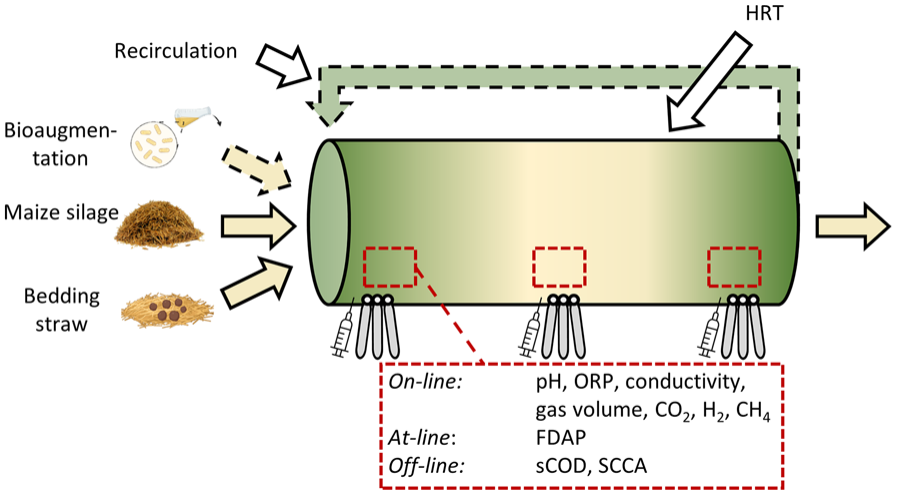
Schematic overview of this study about plug-flow digestion under dynamic conditions with *on-line* gradient monitoring

Two steel PFRs (PFR1, PFR2) were equipped with a set of three *on-line* sensors of the pH-value (EasyFerm Plus PHI Arc 120), conductivity (Conducell 4USF Arc 120) and ORP (EasyFerm Plus ORP ARC 120, all sensors from Hamilton Bonaduz AG, Switzerland)—each with integrated temperature measurement. These sensor triplets were installed at three locations along the horizontally operated PFR, namely, at the inlet, center and outlet (see Fig. 1 and Menzel et al. 2023a). *On-line* measurement of the gas phase was conducted in one of the reactors (PFR2) using BCP-CO_2_, BCP-H_2_, BCP-CH_4_ sensors and a BlueVcount gas volume measurement device (BlueSens gas sensor GmbH, Germany). Process performance was evaluated by the measurement of metabolites (sCOD, SCCA) and by the *at-line* determination of the physiological state of the mixed culture measured by FDAP. The efficiency of hydrolysis and acidogenesis was evaluated by calculation of the specific hydrolysis and acid yield and their corresponding conversion rates (Menzel et al. 2023b).

Monitoring of gradients in two parallel PFRs was applied for the mesophilic digestion of pure MS and MS mixed with 30% or 66% (w/w) of bedding straw under different operation conditions of HRT, thin-sludge recirculation and bioaugmentation, as shown in Table 1, and described previously (Menzel et al. 2023a; b).

**Table 1:** Process conditions applied in the continuous operation of two parallel PFRs

Substrate	HRT (d)	Recirculation	Bioaugmentation	Period of operation (w)
PFR1				
Maize silage	Start-up	–	–	1–3
	7			9–11 12–14
		20%		
	14	–		4^a^–8 15–16/21–22
		20%	*Paenibacillus* spp.	17–20/23–26
			–	27–30
30% straw, MS				31–35.5 36–40/43–46
			*Paenibacillus* spp.	
		–	–	41–42
66% straw, MS		20%		47–54 55–60/64–69
			*Paenibacillus* spp.	
		10% 0%	–	70–74 75–78/61–62
PFR2				
Maize silage	Start-up	–	–	1–4
	14			5^a^–8 9–13
		20%		
Process in PFR2 stopped. Restart after repair				
30% straw, MS	Start-up	–	–	20
	14	20%		21–26 27–32
	21			
66% straw, MS		20%		34–45 46–51
	14			

^a^Base addition for pH-adjustment, see (Menzel et al. 2023b)

### Data evaluation and correlation analysis

*On-line* data was measured in 10 min intervals over the whole measurement period. To correlate the *on-line* parameters to the *at-* and *off-line* measurement of metabolites (measured twice per week), the weekly average of all parameters was used for further calculations. The three-dimensional data acquired at four frequencies (200, 400, 900, 2100 kHz) from the FDAP measurement was transformed by principal component analysis to two principal component (PC) scores, where PC1 is mainly describing the variation of FDAP over time and PC2 the signal’s slope among the frequencies (Longis et al. 2023).

The weekly averages of all parameters and the PC scores were normalized [0,1] and linear Pearson correlation was conducted using Origin(Pro) 2015 (OriginLab Corp., MA). To further identify possible non-linear relationships between *on-line* and *off-line* measurements, their dependencies were plotted using SigmaPlot (version 12.5, Systat Software Inc., IL).

## Results and discussion

### Gradient development under dynamic long-term operation

**Fig. 2:**
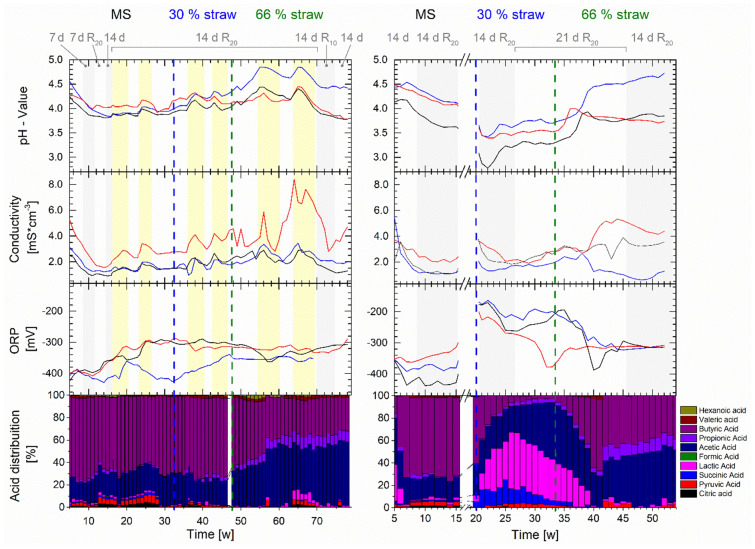
Development of gradients of the pH-value, conductivity and ORP between the inlet (blue), center (red) and outlet (black) ports in PFR1 (left column) and PFR2 (right column) under dynamic conditions. A substrate change is indicated by a dashed line, changes in HRT or thin-sludge recirculation (*R*) are marked in grey/white and phases of bioaugmentation are marked in yellow. Changes of the microbial community and the acidogenic metabolism are indicated by the variations in the distribution of the produced SCCA

The main purpose of investigations was the identification of any correlations between local and gradient measurements in microbial hydrolysis in a PFR. As expected, in both reactors, gradients of the pH-value, the conductivty and the ORP developed over the length of the reactors and over time, as shown in Fig. 2. This gradient development should usually be a good indicator for a dominant plug-flow regime and emphasizes the phase formation (first a dominant hydrolysis, second a dominant acidogenesis) within the reactors, as described in the “Variation of thin-sludge recirculation, feedstock composition and HRT” section.

The dynamic process operation induced changes in the scale and order of the gradients. In particular, changes of the feedstock composition, the SCCA composition and bioaugmentation were found to influence the spatial gradient development in the PFRs. In contrast, variation of the HRT or thin-sludge recirculation did not provoke significant changes in the gradient formation. The most stable gradient between ports was found for the conductivity, where the center values were, on average, higher by 1779 and 835 µS cm^−1^ than values that were measured at the in- and outlet port in PFR1 and during hydrolysis of MS in PFR2 (Fig. 2). This can be regarded as significant as even after the whole experiment, sensor deviations of up to − 300 µS cm^−1^ were detected, prooving the significance of this gradient. The gradient of conductivity was influenced by the basal conductivity of the bedding straw, leading to spikes and high values, espacially in the center, when a higher straw content was applied in the feed. Bioaugmentation with hydrolytic *Paenibacillus* spp. led to a further increase of conductivity in the center (Menzel et al. 2023a). These local increments were related to a higher release of SCCA in the center of the PFRs, as discussed in the “Variation of thin-sludge recirculation, feedstock composition and HRT” section. The described conductivity gradient was mostly accompanied with a stable microbial metabolism as butyric and acetic acid accumulated constantly. An ORP below − 300 mV was measured among all ports. It was described before that the ORP is related with the dominant metabolic routes, where values between − 250 to − 300 mV led to maximal butyric acid accumulation (Chen et al. 2015). Transition phases with an altering SCCA profile as found in PFR2, occurred together with a changed conductivity gradient and an increasing ORP measurement of between -200 and − 300 mV. This can be regarded as significant as the highest deviance of measurement signals from calibration solutions after sensor utilisation was − 35 mV. When the microbial community switched back to the dominant production of butyric and acetic acid in week 40 in PFR2 (Fig. 2), the ORP stabilized between − 300 and − 310 mV among all ports when also a maximal conductivity was measured in the center. Microbial or metabolic changes and instabilities in the reactor might thus be recognizable using gradient monitoring of these parameters, which is further examined by correlation analyses (“Variation of thin-sludge recirculation, feedstock composition and HRT” and “Robustness of spatial on-line monitoring” sections).

The feedstock had a high influence on the pH-gradient in the reactors: a higher straw content led to a more pronounced pH-gradient that decreased from the inlet to the end of the reactor. Acid production in the center and outlet of the PFRs reduced the pH-value along the reactor. However, the high inherent acidity of MS disturbed this gradient development in PFR1 during MS digestion, while it was still found in PFR2. A different feedstock composition may change the buffer capacity. Therefore, any gradient formation in this zone might also depend on this factor, however, the separation of effects is difficult or even impossible. Bioaugmentation mostly induced a global increase of the pH-value, likely caused by a higher release of alkaline substances originating from the substrate due to increased hydrolytic activity (Menzel et al. 2023a). Since pH-values never exposed a higher deviance than 0.1 to calibration solutions, gradients can be regarded as significant.

### Variation of thin-sludge recirculation, feedstock composition and HRT

**Fig. 3:**
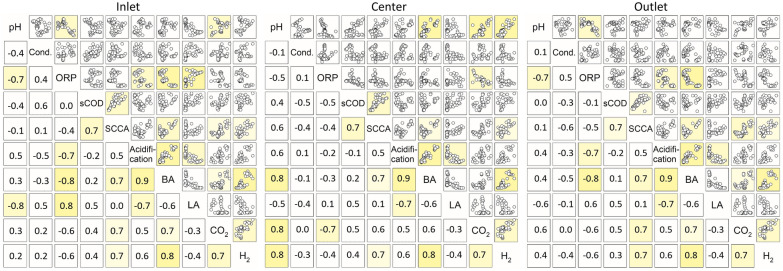
Linear Pearson correlation of the *on-line* measurements with metabolite concentrations in PFR2 at the inlet, center and outlet port. Depicted are the weekly average values from 53 weeks of operation with different feedstock, HRT variation, changes in microbial metabolism and under thin-sludge recirculation. Correlation coefficients ≥ 0.7 are marked for visualization of the location-dependent correlation pattern*;* *SCCA* short-chain carboxylic acids, *BA* butyric acid, *LA* lactic acid

To better understand the dependencies between *on-line* measurements and the overall process performance, further analysis was conducted with respect to values from the spatially distributed measurement spots, namely, the inlet, center and outlet part of the reactor. The measurements revealed several relations to the actual process performance. First, the *on-line* data of liquid and gas sensors was correlated to the main metabolites’ accumulation in the reactor as shown in Fig. 3. Further correlation analysis for all parameters was performed. The correlation coefficients are listed in Additional file 1: Table S1.

Several correlations were found among the metabolites’ accumulation among all ports, where the accumulation of butyric acid was related to a high acidification, total SCCA concentration, H_2_ and CO_2_ production, and correspondingly to an increased acid yield and acid production rate. This is in accordance with the typical pathway of butyric acid production in AD, where butyric acid release is connected to H_2_ and CO_2_ production (Luo et al. 2019). Moreover, a high dependency of the acidogenesis on hydrolysis was found, with linear correlations of the SCCA concentration to the sCOD, and the acid yield and production rate to the hydrolysis yield and rate. This is to be expected, as a higher availability of soluble metabolites should increase the acid production and yield. The linearity further shows that acidogenesis was not inhibited by product accumulation, but limited by a slow substrate hydrolysis. The bioaugmentation experiments in PFR1 led to outliers from this linear dependence: during the addition phase of hydrolytic microorganisms, the acid yield increased while the hydrolysis yield remained unchanged or decreased (Menzel et al. 2023a). This was detected with both straw-containing feedstock compositions in particular, whereas the correlation remained linear for MS. Bioaugmentation increased the acid yield from straw by enhancing the conversion of already soluble polymeric molecules into SCCAs, but could not or merely increase the actual solubilization of particulate and recalcitrant straw substrate (Menzel et al. 2023a).

As previously stated (Menzel et al. 2023b), different fermentation patterns directed to butyric or lactic acid were found in PFR2, where lactic acid production is correlated to a low acidification (− 0.7) and not related to gas production. The *on-line* monitoring revealed that changes in the accumulating SCCAs (that is a different dominant fermentation pattern) can be recognized using gradient monitoring. A low pH-value and higher ORP measurements at the inlet favor lactic acid fermentation, whereas a higher pH-value at the center combined with a low ORP at the inlet and outlet results in a dominant butyric acid fermentation. Keeping the inlet pH-value above the threshold for lactic acid fermentation [around 4.0 (Esquivel-Elizondo et al. 2017; Detman et al. 2021)] and a low ORP (≤ − 250 mV) in the inlet seems suitable to prevent lactic acid accumulation. It obviously occurs mostly at the inlet zone due to its close relation to the pH-value (− 0.8) and the ORP (0.8). Moreover, lactic acid bacteria growth is favored on easily degradable substrates like hexose sugars (e.g., from starch of MS) (Chatellard et al. 2016). Their availability is comparably high at the inlet port, as they are hydrolyzed more quickly, and thus exhausted in the rear part of the PFR in comparison to recalcitrant substrate like cellulose from straw (Lindner et al. 2016).

A negative correlation of the ORP with the acidification and butyric acid concentration, and conversely for lactic acid, was found at the inlet port. As butyric and lactic acid are rather inversely related, the ORP measurement was not related to the total SCCA concentration measurement. Hence, in this case, correlations exist under particular, but not all dominant metabolic fermentation patterns during the course of experiments.

The overall correlation patterns between *on-* and *off-line* measurements were quite similar for the in- and outlet (see Fig. 3). In contrast, a distinct pattern of different correlations was found for the center part. There, the ORP was related to CO_2_ production, and the pH-value was correlated with butyric acid, H_2_ and CO_2_. This led to the conclusion, that most likely a major part of acidogenesis took part at the center of the reactor, while at the in- and outlet, hydrolytic processes (or lactic acid fermentation at the inlet) dominated over acidogenic processes. Similarities between in- and outlet were presumably further intensified by thin-sludge recirculation. A corresponding enrichment of functional microorganisms in these phases is highly probable, but requires further microbial community analysis for a final confirmation.

Similar patterns have been found before in PFRs. Longis et al. (2023) found good correlations of the pH-value and the conductivity with H_2_, CO_2_ and SCCA accumulation in the center of a PFR in acidic AD, while those were minor at the inlet and outlet. The emergence of two hydrolytic phases was also observed during full AD in PFRs (Dong et al. 2019; Rossi et al. 2022). Dong et al. (2019) observed an enrichment of hydrolytic bacteria in the inlet and cellulolytic bacteria (*Christensenellaceae* sp., *Ruminofilibacter* sp., among others) in the outlet part of a PFR when operated with cattle manure. During the thermophilic AD of organic municipal waste, Rossi et al. (2022) found similar microbial communities with hydrolytic activity (*Thermotogae* sp.) at the inlet and outlet part of a PFR that was operated with recirculation, whereas the center showed a higher abundance of acidogenic *Clostridia* spp. Most likely, as also proposed by Dong et al. (2019), rather easily convertible substrate is hydrolyzed in the inlet part and further metabolized in the center. Once exhausted, hydrolytic activity increases again in the back part of the reactor for the breakdown of more recalcitrant substrate.

No direct correlations of the *on-line* measurements were found with the hydrolytic process efficiency, expressed as specific hydrolysis yield/rate (derived as described in Menzel et al. (2023b), correlation values are shown in Additional file 1). The diversity of hydrolysis metabolites and their conversion into acids make it challenging to detect potential correlations with such intermediates. However, butyric acid production was closely related to a high acid yield and production rate (Pearson *R* = 0.8/0.7, see Additional file 1). Based on the relation of the pH-value at the center and the ORP at the inlet to butyric acid, we discovered an indirect measurement of the acid yield. A multi-linear regression model of the expected acid yield can be achieved from *on-line* measurements under dynamic conditions and changing feedstock.

Local monitoring in the PFR system can support the control of fermentation patterns in acidogenic digestion and thereby the acid and hydrogen yield, or foster lactic acid accumulation. Moreover, the phase formation of the PFR into two hydrolytic and one acidogenic phase was found, showing that a spatial emergence of dominant fermentation phases with specialized microbial communities occurs.

### Variation of thin-sludge recirculation, HRT and bioaugmentation at static feedstock composition

Before, the value of multiposition *on-line* monitoring under the operation with different feedstock composition was described. However, the metabolism and activity of hydrolytic and acidogenic microorganisms is also highly dependent on other factors. Therefore, and for the relevance in practice, we examined correlations at variations of the HRT, recirculation and bioaugmentation while the feedstock composition remained unchanged, to identify *on-line* parameters that are suitable for process control under those conditions.

**Fig. 4:**
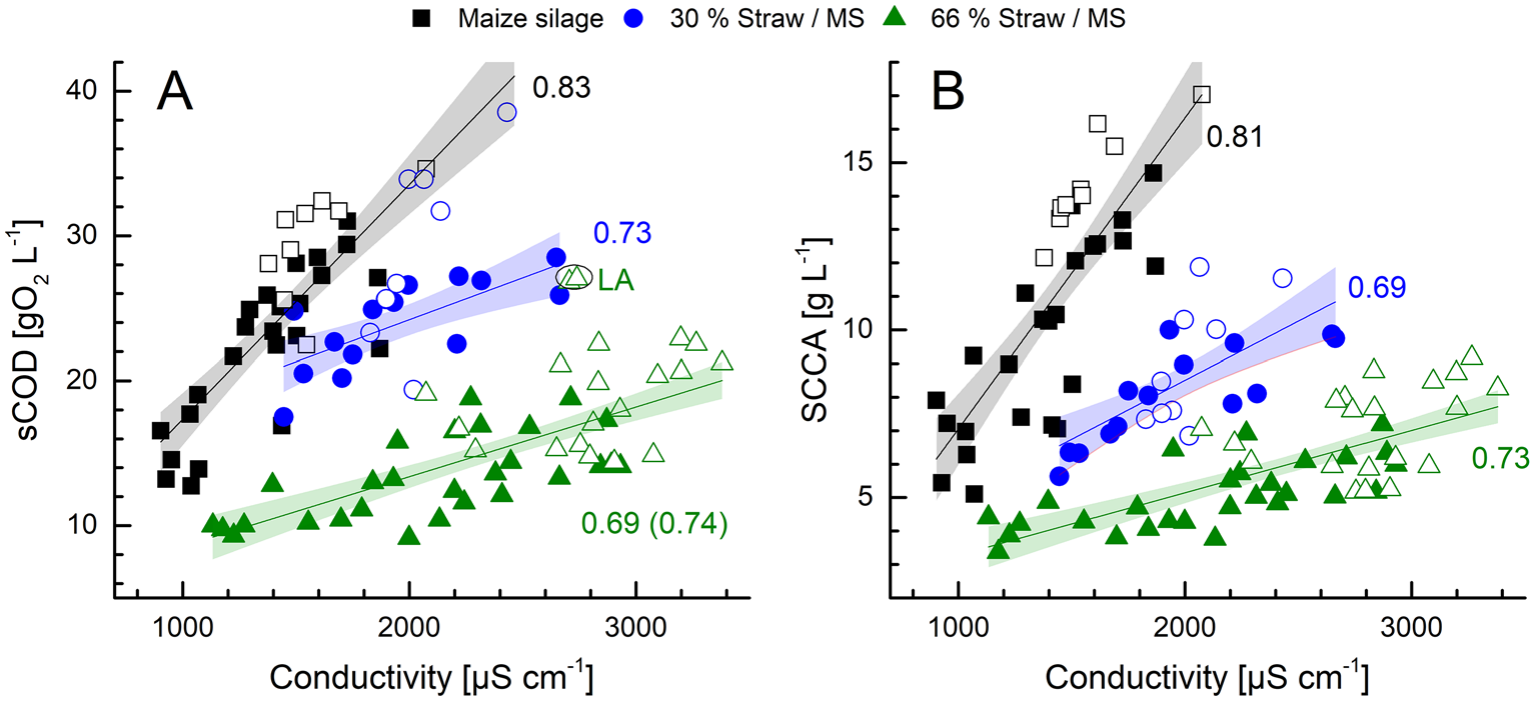
Linear Pearson correlation with 90% confidence band and corresponding Pearson coefficient of the conductivity at the outlet port with sCOD (**A**) and SCCA (**B**) depending on the feedstock for PFR1 (filled symbols) and PFR2 (open symbols). Each symbol represents the average measurement of one week. Measurements during start-up (W1–W6) and the first HRT after a feedstock change were excluded. The data points for PFR2 sCOD-conductivity with dominant lactic acid fermentation with 30% straw (w/w) are not included in the linear correlation. For 66% straw (w/w) sCOD-conductivity, weeks with dominant lactic acid fermentation are marked (LA) and the correlation coefficient excluding these points is presented in brackets, *R*^2^ values can be found in Additional file 1: Table S3

In particular, the measurement of the conductivity at the outlet shows a good linear correlation with the concentration of sCOD and SCCAs depending on the feedstock composition as presented in Fig. 4. The correlation of the sCOD and the conductivity expresses different linear slopes in dependence on the acid metabolism, whether it is directed towards lactic acid or butyric acid (see Fig. 4A; PFR2 with dominant lactic acid, PFR1 with dominant butyric acid fermentation). A dominant lactic acid fermentation showed a higher sCOD concentration than a dominant butyric acid fermentation while conductivity measurements remained similar. The hydrolysis was increased by 17% during lactic acid fermentation (Menzel et al. 2023b). The correlation of the SCCA concentration and conductivity was more profound (Pearson *R* ≥ 0.7) even under different states of acid metabolisms. The *on-line* measurement of conductivity at the outlet can thus be used to predict acid concentrations in the outlet part of the PFR during dynamic operation conditions. As these correlations were found in good alignment for both reactors and experiments, transferability is assumed.

A linear correlation of the conductivity with acid concentration in acidic AD has been found before in stirred tank reactors (Aceves-Lara et al. 2012; Marín-Peña et al. 2020). The application of a spatial monitoring strategy using *on-line* sensors at three ports of a PFR showed good correlations of the conductivity with SCCA concentrations at the inlet and center, while there was no correlation at the outlet likely due to their re-assimilation (Longis et al. 2023). However, in our study, higher fluctuations of conductivity due to the feedstock or bioaugmentation were found in the inlet or center port, thereby weakening the correlation, whereas the outlet conductivity showed a good fit. The lower pH-values in this study, compared to Longis et al. (2023), might have prevented the re-assimilation of acids at the outlet. In addition, the HRT might be more suitable to maintain a surplus of SCCA release along the whole PFR.

The monitoring of the conductivity, especially at the outlet, proved to be a reliable indicator for the accumulation of the total SCCAs, in both PFRs and under significant operational (HRT, recirculation) and microbial/process changes (bioaugmentation, variation in metabolism).

**Fig. 5:**
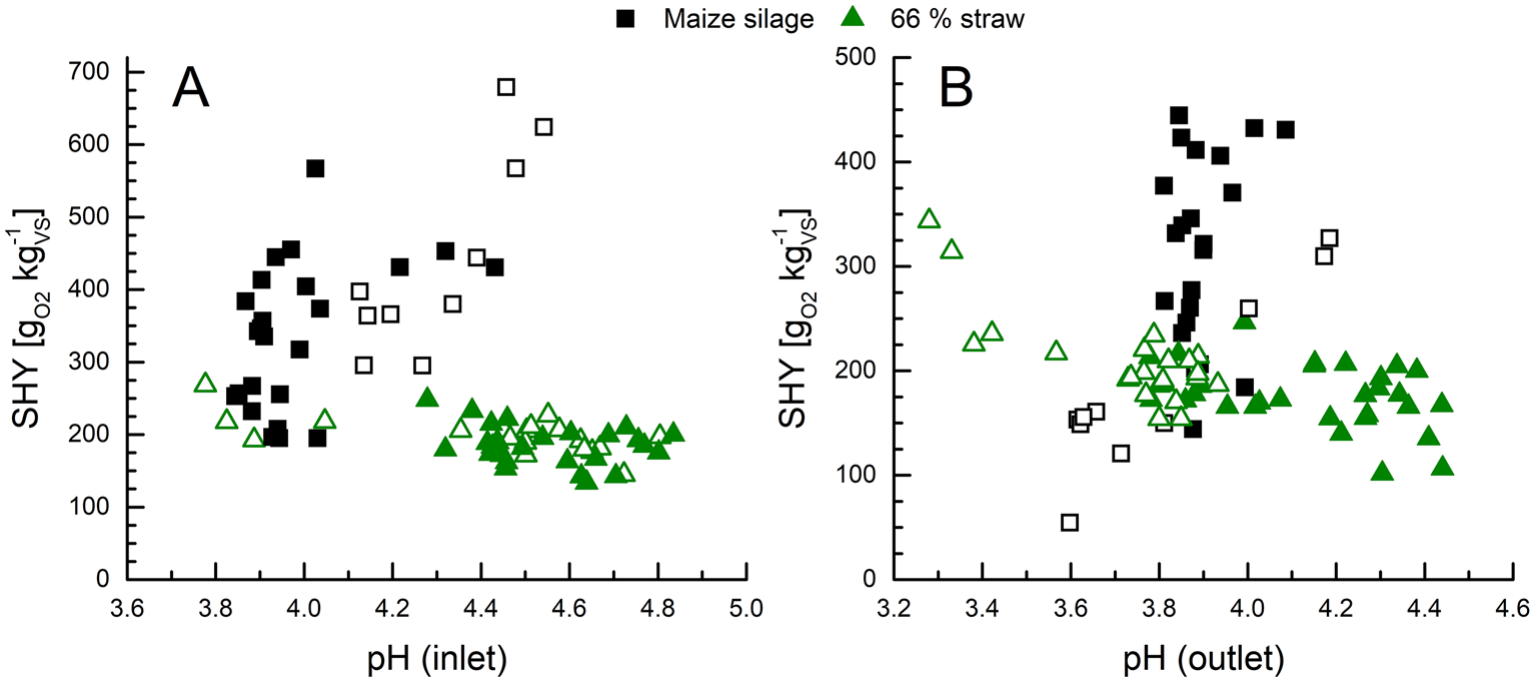
Feedstock-related dependance of the specific hydrolysis yield (SHY) on the pH-value at the inlet (**A**) and outlet (**B**) port for PFR1 (filled symbols) and PFR2 (open symbols). Measurements during start-up (W1–5) and the first 2 weeks with 7 d of HRT in PFR1 (weeks 9, 10) were excluded

Although no general correlation of the *on-line* parameters to the hydrolytic performance (rate or yield) was found, the pH-value seems to be of importance. Contrary relations of the specific hydrolysis yield and the pH-value in the inlet and outlet were seen (Fig. 5). For MS, a higher pH-value was found to generally increase the hydrolysis yield. This is caused by the higher activity of hydrolytic microorganisms at less acidic pH-values (Azman et al. 2015). A reverse trend was found, when the feedstock consisted of 66% of bedding straw, a highly lignocellulosic biomass. There, the maximum hydrolysis yield correlated with very low pH-values at the in- and outlet, whereas an increase in pH rather resulted in a reduced hydrolysis. In our study, the pH-range of between 3.2 and 4.9 was very low for the microbial digestion of cellulose. Most anaerobic cellulolytic bacteria can efficiently digest cellulose at rather neutral pH-values between 6.4 and 7.1 (Desvaux 2005; Zverlov et al. 2010). While microbial hydrolysis of cellulose was eventually hindered, it seems that the lignocellulosic structures of the straw were partially loosened by the high acidity. The presence of SCCA, such as acetic acid, which accumulated during acidogenesis, has been demonstrated to induce swelling and fragmentation of lignocellulosic biomass. This process leads to an enlargement of the surface area and higher porosity, which subsequently exposes hemicellulose and cellulose to enzymatic degradation (Peng et al. 2019; Ma et al. 2021). In the acidogenic co-digestion of corn cob and food waste, about 10% of the crystalline cellulose were dismantled at pH 4.0 (Zou et al. 2020). This kind of acidic pre-treatment also increased hydrolysis in our study and thereby demonstrated, that the acidic digestion of lignocellulosic residues at pH-values below 4.0 is feasible to produce lactic acid in PFRs.

### Robustness of spatial on-line monitoring

Local monitoring in a hydrolytic plug-flow system was shown to be of high value for the *on-line* determination of fermentation patterns or acid concentrations. However, an *on-line* monitoring system for practical use should show a high robustness towards both, changing feedstock or other process conditions. Thus, we evaluated the overall data collected from the two PFRs during their continuous operation (exclusion of start-up phase: weeks 1–6) for a total of 123 weeks to identify the most robust and versatile sensor parameters and positions within this system.

**Fig. 6:**
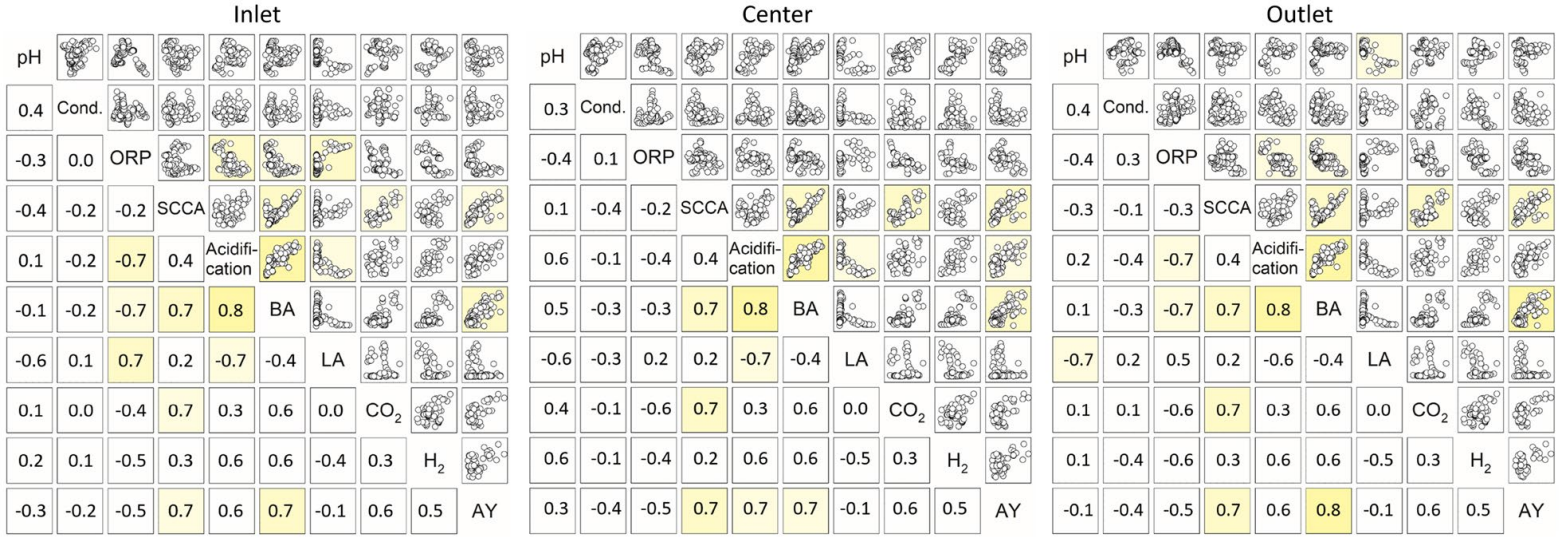
Linear Pearson correlation analysis of *on-line* measurements with parameters related to acidogenesis from both PFRs during continuous fermentation over 123 weeks (weekly average values). Correlation coefficients ≥ 0.7 are marked for visualization of the location-dependent correlation pattern*.* Excluded data points: start-up phase (W1–W6, W20–PFR2), first week of 7 d HRT (W9–PFR1). *SCCA* short-chain carboxylic acids, *BA* butyric acid, *LA* lactic acid, *AY* acid yield

Overall, *on-line* monitoring was connected to acidogenesis, but showed no correlation with the hydrolytic performance in the PFR (see Additional file 1: Table S2). This might be due to the fact that microbial hydrolysis itself was influenced negatively at some feedstock compositions by the low pH-values between 3.2 and 4.9 as mentioned previously. As described in the “Variation of thin-sludge recirculation, feedstock composition and HRT” section, the ORP measurement at the inlet showed a direct correlation with the acidification, butyric acid and lactic acid concentration (see Fig. 6). *On-line* measurement of the pH-value and the conductivity were less relevant for quantifying acidification. The SCCA concentration itself was also related to the CO_2_ concentration, which shows that off-gas measurement can be used for the indirect determination of the SCCA concentration. In combination with the ORP measurement at the inlet, even the identification of fermentation patterns either towards butyric or lactic acid dominance might be possible. The relation of butyric acid to hydrogen production did, however, not remain constant in both reactors. This might indicate the presence of hydrogen-consuming pathways (e.g., homoacetogenesis, lactic/propionic acid production) or the presence of other butyric acid producing pathways, which impact hydrogen release differently. The direct conversion of carbohydrates to butyric acid is connected with the release of hydrogen and often performed by microorganisms of the genus *Clostridium*, *Bacillus* or *Bacteroides* (Luo et al. 2019) However, interconversion reactions from acetic, lactic or succinic acid to butyric acid with different molar ratios of hydrogen release have also been found to be of high relevance during the acidic AD of carbohydrates (Esquivel-Elizondo et al. 2017; Detman et al. 2021; Brodowski et al. 2022). The complex combination of competing metabolic pathways during dynamic conditions in the PFRs apparently impaired the application of hydrogen *on-line* measurement for the detection of butyric acid.

Overall, the ORP measurement at the inlet proved to be suitable. Combined with the concurrent monitoring of the *on-line* CO_2_ production, a soft sensor for the *on-line* determination of acid yield and SCCA concentration can be obtained.

## Conclusion

The long-term dynamic hydrolytic digestion in PFRs was examined using multiposition *on-line* monitoring of the pH, conductivity and the ORP. Phase formation inside the PFRs was confirmed with a dominant hydrolytic metabolism at the in- and outlet, lactic acid fermentation at the inlet (pH < 4.0 and ORP − 200 to − 300 mV) and acidogenesis with dominant butyric acid accumulation in the center (ORP < − 300 mV). Direct correlations between the acidogenic process performance (dominant fermentation patterns) and the pH-value (inlet/center), ORP (inlet/center) and conductivity (outlet, feedstock dependent) were found. Individual measurements at these spots had a greater relevance for any correlation with *off-line* parameters than the consideration of gradients. The presented *on-line* multiposition monitoring strategy opens further possibilities for large-scale control of plug-flow microbial hydrolysis under dynamic process conditions. Further work will, beside an application in larger scales, also include multi-factor analysis, probably on a wider data set, to further contribute to the evaluation of the applied monitoring strategy.

### Supplementary Information

**Additional file 1: Table S1.** Pearson correlation factors between all measured parameters for PFR2 during W1—W53 (week 5 excluded) at the inlet (top, white) and center (grey), and outlet port (bottom, white) of the reactor. **Table S2.** Pearson correlation factors between *on-line* monitoring and measured process parameters for both PFRs in the inlet (top, white), center (grey), and outlet port (bottom, white) during continuous dynamic fermentation over 123 weeks, with variations of feedstock composition, HRT, thin-sludge-recirculation and under bioaugmentation. **Table S3.** Correlation coefficients and R^2^ values for the relation of outlet-conductivity to sCOD and SCCA (Fig. 4).

## Data Availability

The data set supporting the conclusions of this article is available at DOI: https://doi.org/10.14279/depositonce-19027 and within Additional file 1: Table S1–3. The full data are available from the corresponding authors on reasonable request.

## Abbreviations

AD: Anaerobic digestion
FDAP: Frequency-dispersed anisotropy polarizability
HRT: Hydraulic retention time
MS: Maize silage
ORP: Oxidation–reduction potential
PC: Principal component
PFR: Plug-flow reactor
sCOD: Soluble chemical oxygen demand
SCCA: Short-chain carboxylic acid
