# Supplementary material for: Spatial monitoring of hydrolysis in a plug-flow bioreactor: a support for flexible operation?
Source: Bioresour Bioprocess. 2024 Feb 14;11(1):23. doi: 10.1186/s40643-024-00740-0 (PMC10992403; doi:10.1186/s40643-024-00740-0)
Supplement: Supplementary file 1 — Additional file 1: Table S1. Pearson correlation factors between all measured parameters for PFR2 during W1—W53 (week 5 excluded) at the inlet (top, white) and center (grey), and outlet port (bottom, white) of the reactor. Table S2. Pearson correlation factors between on-line monitoring and measured process parameters for both PFRs in the inlet (top, white), center (grey), and outlet port (bottom, white) during continuous dynamic fermentation over 123 weeks, with variations of feedstock composition, HRT, thin-sludge-recirculation and under bioaugmentation. Table S3. Correlation coefficients and R2 values for the relation of outlet-conductivity to sCOD and SCCA (Fig. 4). [file 40643_2024_740_MOESM1_ESM.docx]

Additional material

Spatial monitoring of hydrolysis in a plug-flow bioreactor: a support for flexible operation?

Theresa Menzel^1^[
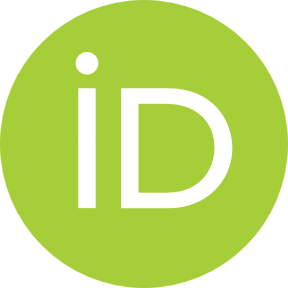
](https://orcid.org/0000-0003-3044-9784), Peter Neubauer^1^[
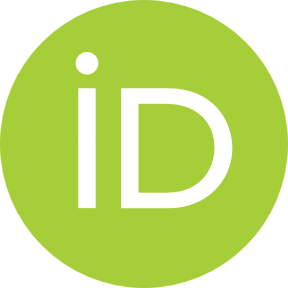
](https://orcid.org/0000-0002-1214-9713) and Stefan Junne^1,2^[
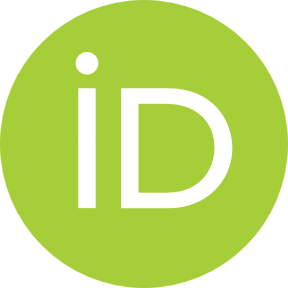
](https://orcid.org/0000-0001-6185-2627) *

1) Chair of Bioprocess Engineering, Institute of Biotechnology, Technische Universität Berlin, Ackerstraße 76, ACK 24, D-13355 Berlin, Germany; t.menzel@tu-berlin.de (T.M.); peter.neubauer@tu-berlin.de (P.N.)

2) Department of Chemistry and Bioscience, Aalborg University Esbjerg, Niels Bohrs Vej 8, DK-6700 Esbjerg, Denmark

* Corresponding author: sju@bio.aau.dk

**Table S1** Pearson correlation factors between all measured parameters for PFR2 during W1 - W53 (week 5 excluded) at the inlet (top, white) and center (grey), and outlet port (bottom, white) of the reactor

|  | *pH* | *Cond.* | *ORP* | *sCOD* | *SCCA* | *Acidifi-cation* | *PCA 1 (FDAP)* | *PCA 2 (FDAP)* | *SHY* | *SHR* | *AY* | *APR* | *Butyric acid* | *Lactic acid* | *CO_2_* | *H_2_* | *CH_4_* | *Gas yield* |
| --- | --- | --- | --- | --- | --- | --- | --- | --- | --- | --- | --- | --- | --- | --- | --- | --- | --- | --- |
| *pH* |  | -0.37 | **-0.72** | -0.41 | -0.14 | 0.47 | 0.22 | -0.58 | -0.04 | 0.12 | 0.02 | 0.18 | 0.34 | **-0.78** | 0.32 | 0.21 | -0.19 | 0.31 |
| *Cond.* | -0.10 |  | 0.39 | 0.58 | 0.12 | -0.47 | 0.02 | 0.02 | 0.08 | 0.03 | 0.00 | -0.10 | -0.26 | 0.49 | 0.20 | 0.15 | **0.81** | 0.25 |
| *ORP* | -0.55 | 0.08 |  | 0.02 | -0.41 | **-0.73** | -0.14 | 0.47 | -0.43 | -0.47 | -0.52 | -0.53 | **-0.79** | **0.75** | -0.57 | -0.63 | 0.34 | -0.43 |
| *sCOD* | 0.39 | -0.51 | -0.48 |  | **0.74** | -0.16 | 0.06 | 0.02 | 0.58 | 0.32 | 0.55 | 0.22 | 0.20 | 0.48 | 0.43 | 0.38 | 0.14 | 0.28 |
| *SCCA* | 0.56 | -0.42 | -0.43 | **0.73** |  | 0.50 | 0.03 | -0.15 | **0.70** | 0.58 | **0.85** | 0.62 | 0.71 | 0.04 | 0.67 | 0.67 | -0.26 | 0.38 |
| *Acidification* | 0.57 | 0.12 | -0.21 | -0.11 | 0.50 |  | 0.01 | -0.29 | 0.33 | 0.43 | 0.61 | 0.59 | **0.88** | **-0.71** | 0.54 | 0.64 | -0.43 | 0.25 |
| *PCA1 (FDAP)* | 0.07 | -0.03 | -0.16 | -0.09 | -0.16 | -0.06 |  | 0.25 | 0.17 | 0.06 | 0.17 | 0.09 | -0.01 | -0.06 | -0.25 | 0.02 | -0.28 | -0.04 |
| *PCA 2 (FDAP)* | -0.37 | -0.31 | 0.56 | -0.02 | -0.05 | -0.17 | 0.08 |  | -0.13 | -0.16 | -0.03 | -0.11 | -0.30 | 0.35 | -0.62 | -0.27 | -0.31 | -0.43 |
| *SHY* | 0.47 | -0.17 | -0.21 | 0.36 | 0.52 | 0.46 | -0.06 | -0.11 |  | **0.94** | **0.81** | **0.77** | 0.56 | 0.00 | 0.41 | 0.55 | -0.07 | 0.36 |
| *SHR* | 0.62 | -0.40 | -0.28 | 0.54 | **0.86** | 0.64 | -0.12 | 0.08 | **0.93** |  | **0.86** | **0.92** | 0.61 | -0.21 | 0.46 | 0.60 | 0.02 | 0.43 |
| *AY* | 0.43 | -0.16 | -0.18 | 0.24 | 0.60 | 0.62 | -0.03 | -0.05 | **0.78** | **0.81** |  | **0.95** | **0.79** | -0.17 | 0.56 | 0.68 | -0.33 | 0.37 |
| *APR* | **0.77** | -0.12 | -0.28 | 0.23 | 0.68 | **0.90** | -0.06 | -0.16 | **0.73** | **0.89** | **0.94** |  | **0.69** | -0.30 | 0.53 | 0.65 | -0.13 | 0.42 |
| *Butyric acid* | **0.77** | -0.12 | -0.28 | 0.23 | 0.68 | **0.90** | -0.06 | -0.16 | 0.62 | 0.62 | **0.78** | **0.69** |  | -0.63 | 0.66 | **0.78** | -0.38 | 0.34 |
| *Lactic acid* | -0.47 | -0.43 | 0.08 | 0.46 | 0.10 | **-0.70** | -0.21 | 0.22 | -0.04 | -0.22 | -0.13 | -0.28 | -0.62 |  | -0.26 | -0.38 | 0.32 | -0.14 |
| *CO_2_* | **0.75** | 0.03 | **-0.72** | 0.49 | 0.64 | 0.54 | -0.12 | -0.51 | 0.48 | 0.51 | 0.55 | 0.52 | 0.65 | -0.25 |  | **0.69** | 0.06 | 0.63 |
| *H_2_* | **0.81** | -0.27 | -0.38 | 0.41 | 0.68 | 0.64 | -0.03 | -0.14 | 0.60 | 0.63 | **0.71** | 0.68 | **0.78** | -0.37 | **0.69** |  | 0.08 | 0.58 |
| *CH_4_* | -0.09 | 0.60 | 0.09 | 0.03 | -0.22 | -0.43 | -0.10 | -0.21 | -0.14 | -0.01 | -0.32 | -0.13 | -0.40 | 0.34 | 0.06 | 0.08 |  | 0.34 |
| *Gas yield* | 0.56 | 0.06 | -0.41 | 0.31 | 0.38 | 0.25 | 0.04 | -0.33 | 0.37 | 0.45 | 0.39 | 0.45 | 0.35 | -0.13 | 0.63 | 0.58 | 0.34 |  |
|  |  |  |  |  |  |  |  |  |  |  |  |  |  |  |  |  |  |  |
| *pH* |  | 0.09 | **-0.70** | -0.02 | 0.09 | 0.40 | 0.12 | -0.56 | 0.21 | 0.22 | 0.19 | 0.19 | 0.43 | -0.61 | 0.56 | 0.41 | -0.03 | 0.52 |
| *Cond.* |  |  | 0.48 | -0.31 | -0.56 | -0.27 | -0.07 | -0.18 | -0.41 | -0.17 | -0.60 | -0.27 | -0.46 | -0.13 | -0.04 | -0.38 | **0.71** | 0.08 |
| *ORP* |  |  |  | -0.06 | -0.50 | **-0.72** | -0.08 | 0.39 | -0.42 | -0.40 | -0.59 | -0.47 | **-0.82** | 0.58 | -0.59 | -0.63 | 0.44 | -0.44 |
| *sCOD* |  |  |  |  | 0.67 | -0.20 | 0.05 | -0.21 | **0.74** | 0.38 | 0.47 | 0.22 | 0.15 | 0.48 | 0.48 | 0.32 | 0.13 | 0.31 |
| *SCCA* |  |  |  |  |  | 0.47 | 0.04 | -0.22 | **0.75** | 0.57 | **0.87** | 0.64 | **0.72** | 0.06 | **0.69** | 0.68 | -0.25 | 0.38 |
| *Acidification* |  |  |  |  |  |  | 0.01 | -0.18 | 0.25 | 0.38 | 0.60 | 0.59 | **0.87** | **-0.70** | 0.54 | 0.64 | -0.43 | 0.25 |
| *PCA1 (FDAP)* |  |  |  |  |  |  |  | 0.27 | 0.01 | 0.03 | 0.04 | 0.05 | 0.01 | -0.05 | -0.08 | -0.03 | -0.12 | 0.03 |
| *PCA 2 (FDAP)* |  |  |  |  |  |  |  |  | -0.22 | -0.24 | -0.09 | -0.15 | -0.23 | 0.16 | -0.58 | -0.21 | -0.21 | -0.37 |
| *SHY* |  |  |  |  |  |  |  |  |  | **0.94** | **0.86** | **0.84** | 0.56 | 0.04 | 0.59 | 0.58 | -0.03 | 0.46 |
| *SHR* |  |  |  |  |  |  |  |  |  |  | **0.85** | **0.94** | 0.59 | -0.19 | 0.56 | 0.59 | 0.05 | 0.50 |
| *AY* |  |  |  |  |  |  |  |  |  |  |  | **0.95** | **0.80** | -0.17 | 0.59 | 0.69 | -0.32 | 0.38 |
| *APR* |  |  |  |  |  |  |  |  |  |  |  |  | **0.71** | -0.30 | 0.56 | 0.66 | -0.13 | 0.44 |
| *Butyric acid* |  |  |  |  |  |  |  |  |  |  |  |  |  | -0.61 | 0.67 | **0.79** | -0.38 | 0.35 |
| *Lactic acid* |  |  |  |  |  |  |  |  |  |  |  |  |  |  | -0.26 | -0.39 | 0.30 | -0.14 |
| *CO_2_* |  |  |  |  |  |  |  |  |  |  |  |  |  |  |  | **0.69** | 0.06 | 0.63 |
| *H_2_* |  |  |  |  |  |  |  |  |  |  |  |  |  |  |  |  | 0.08 | 0.58 |
| *CH_4_* |  |  |  |  |  |  |  |  |  |  |  |  |  |  |  |  |  | 0.34 |

Abbreviations: Cond. - conductivity, ORP - oxidation-reduction potential, sCOD - soluble COD, SCCA - short chain carboxylic acids,
 SHY - specific hydrolysis yield, SHR - specific hydrolysis rate, AY - acid yield, APR - acid production rate

**Table S2** Pearson correlation factors between *on-line* monitoring and measured process parameters for both PFRs in the inlet (top, white), center (grey), and outlet port (bottom, white) during continuous dynamic fermentation over 123 weeks, with variations of feedstock composition, HRT, thin-sludge-recirculation and under bioaugmentation.

|  | *pH* | *Cond.* | *ORP* | *sCOD* | *SCCA* | *Acidifi-cation* | *PCA 1 (FDAP)* | *PCA 2 (FDAP)* | *SHY* | *SHR* | *AY* | *APR* | *Butyric acid* | *Lactic acid* | *CO_2_* | *H_2_* | *CH_4_* | *Gas yield* |
| --- | --- | --- | --- | --- | --- | --- | --- | --- | --- | --- | --- | --- | --- | --- | --- | --- | --- | --- |
| *pH* |  | 0.38 | -0.26 | -0.51 | -0.39 | 0.14 | 0.47 | -0.58 | -0.12 | -0.32 | -0.30 | -0.19 | -0.07 | -0.57 | 0.12 | 0.19 | **0.68** | 0.33 |
| *Cond.* | 0.25 |  | 0.04 | -0.12 | -0.18 | -0.17 | 0.48 | -0.28 | 0.03 | -0.32 | -0.17 | -0.15 | -0.24 | 0.05 | -0.02 | 0.13 | 0.59 | 0.31 |
| *ORP* | -0.40 | 0.08 |  | 0.11 | -0.16 | -0.70 | 0.21 | 0.08 | -0.63 | 0.08 | -0.47 | -0.45 | -0.66 | **0.75** | -0.41 | -0.46 | -0.35 | -0.44 |
| *sCOD* | -0.05 | -0.40 | -0.09 |  | **0.88** | -0.07 | -0.17 | 0.26 | 0.07 | 0.55 | 0.44 | 0.10 | 0.43 | 0.52 | 0.59 | 0.02 | **-0.72** | 0.31 |
| *SCCA* | 0.13 | -0.35 | -0.19 | 0.86 |  | 0.37 | -0.14 | 0.26 | 0.26 | 0.53 | **0.67** | 0.29 | **0.72** | 0.22 | **0.69** | 0.25 | -0.63 | 0.37 |
| *Acidification* | 0.56 | -0.05 | -0.40 | -0.04 | 0.40 |  | -0.09 | 0.02 | 0.55 | 0.05 | 0.60 | 0.49 | **0.79** | **-0.66** | 0.31 | 0.61 | 0.13 | 0.25 |
| *PCA1 (FDAP)* | 0.07 | 0.50 | 0.16 | -0.28 | -0.24 | -0.14 |  | -0.01 | -0.32 | -0.29 | -0.34 | -0.38 | -0.23 | 0.06 | -0.05 | 0.05 | 0.56 | 0.08 |
| *PCA 2 (FDAP)* | -0.27 | -0.25 | 0.34 | 0.24 | 0.23 | 0.01 | 0.01 |  | 0.07 | -0.07 | 0.14 | -0.02 | 0.14 | 0.19 | -0.14 | -0.20 | -0.43 | -0.41 |
| *SHY* | 0.53 | -0.23 | -0.42 | 0.06 | 0.21 | 0.55 | -0.39 | 0.06 |  | 0.22 | 0.81 | **0.83** | 0.53 | -0.39 | 0.03 | 0.59 | 0.35 | 0.41 |
| *SHR* | -0.16 | -0.43 | -0.23 | 0.57 | 0.52 | 0.06 | -0.30 | 0.00 | 0.09 |  | 0.56 | 0.52 | 0.33 | 0.31 | 0.48 | 0.20 | -0.60 | 0.34 |
| *AY* | 0.33 | -0.39 | -0.46 | 0.47 | **0.72** | 0.66 | -0.41 | 0.17 | **0.74** | 0.47 |  | **0.88** | **0.73** | -0.13 | 0.55 | 0.47 | -0.42 | 0.40 |
| *APR* | 0.29 | -0.35 | -0.57 | 0.10 | 0.29 | 0.55 | -0.44 | 0.03 | **0.79** | 0.44 | **0.84** |  | 0.48 | -0.21 | 0.40 | 0.60 | -0.19 | 0.41 |
| *Butyric acid* | 0.52 | -0.26 | -0.30 | 0.45 | **0.71** | **0.80** | -0.27 | 0.12 | 0.50 | 0.34 | **0.75** | 0.51 |  | -0.43 | 0.62 | 0.58 | -0.24 | 0.37 |
| *Lactic acid* | -0.63 | -0.27 | 0.20 | 0.49 | 0.23 | -0.65 | -0.01 | 0.23 | -0.43 | 0.31 | -0.13 | -0.24 | -0.42 |  | -0.01 | -0.42 | -0.50 | -0.18 |
| *CO_2_* | 0.44 | -0.08 | -0.60 | 0.63 | **0.69** | 0.33 | -0.20 | -0.12 | 0.07 | 0.53 | 0.57 | 0.42 | 0.63 | 0.00 |  | 0.33 | -0.18 | 0.64 |
| *H_2_* | 0.57 | -0.07 | -0.40 | 0.00 | 0.24 | 0.62 | 0.04 | -0.11 | 0.62 | 0.21 | 0.48 | 0.61 | 0.58 | -0.45 | 0.30 |  | 0.03 | 0.58 |
| *CH_4_* | 0.40 | 0.66 | -0.03 | **-0.70** | -0.62 | 0.10 | 0.45 | -0.46 | 0.35 | -0.60 | -0.41 | -0.21 | -0.26 | -0.48 | -0.20 | 0.05 |  | 0.34 |
| *Gas yield* | 0.59 | 0.03 | -0.41 | 0.32 | 0.36 | 0.24 | -0.02 | -0.31 | 0.42 | 0.36 | 0.39 | 0.42 | 0.35 | -0.17 | 0.63 | 0.56 | 0.33 |  |
|  |  |  |  |  |  |  |  |  |  |  |  |  |  |  |  |  |  |  |
| *pH* |  | 0.35 | -0.36 | -0.35 | -0.29 | 0.22 | 0.32 | -0.48 | 0.19 | -0.43 | -0.11 | -0.04 | 0.08 | -0.66 | 0.14 | 0.10 | 0.64 | 0.38 |
| *Cond.* |  |  | 0.33 | 0.10 | -0.09 | -0.42 | 0.31 | -0.23 | -0.51 | -0.05 | -0.42 | -0.50 | -0.33 | 0.17 | 0.09 | -0.42 | 0.13 | 0.17 |
| *ORP* |  |  |  | 0.03 | -0.29 | **-0.68** | 0.03 | 0.32 | -0.35 | -0.29 | -0.51 | -0.53 | -0.65 | 0.55 | -0.57 | -0.61 | -0.13 | -0.40 |
| *sCOD* |  |  |  |  | 0.83 | -0.11 | -0.25 | 0.15 | 0.06 | 0.59 | 0.44 | 0.08 | 0.40 | 0.47 | 0.63 | 0.01 | **-0.65** | 0.34 |
| *SCCA* |  |  |  |  |  | 0.38 | -0.27 | 0.12 | 0.19 | 0.54 | **0.71** | 0.30 | **0.72** | 0.19 | **0.70** | 0.27 | -0.64 | 0.36 |
| *Acidification* |  |  |  |  |  |  | -0.19 | -0.04 | 0.50 | 0.00 | 0.64 | 0.55 | **0.78** | -0.64 | 0.26 | 0.61 | 0.10 | 0.19 |
| *PCA1 (FDAP)* |  |  |  |  |  |  |  | -0.04 | -0.38 | -0.28 | -0.43 | -0.43 | -0.29 | -0.04 | -0.16 | 0.02 | 0.45 | 0.05 |
| *PCA 2 (FDAP)* |  |  |  |  |  |  |  |  | 0.05 | -0.02 | 0.08 | -0.01 | 0.04 | 0.29 | -0.25 | -0.23 | -0.38 | -0.34 |
| *SHY* |  |  |  |  |  |  |  |  |  | 0.06 | **0.72** | **0.77** | 0.46 | -0.41 | 0.09 | 0.58 | 0.38 | 0.49 |
| *SHR* |  |  |  |  |  |  |  |  |  |  | 0.50 | 0.47 | 0.31 | 0.36 | 0.60 | 0.17 | -0.63 | 0.47 |
| *AY* |  |  |  |  |  |  |  |  |  |  |  | **0.84** | **0.75** | -0.13 | 0.59 | 0.46 | -0.41 | 0.40 |
| *APR* |  |  |  |  |  |  |  |  |  |  |  |  | 0.51 | -0.24 | 0.45 | 0.60 | -0.18 | 0.43 |
| *Butyric acid* |  |  |  |  |  |  |  |  |  |  |  |  |  | -0.42 | 0.65 | 0.58 | -0.27 | 0.36 |
| *Lactic acid* |  |  |  |  |  |  |  |  |  |  |  |  |  |  | -0.01 | -0.46 | -0.48 | -0.17 |
| *CO_2_* |  |  |  |  |  |  |  |  |  |  |  |  |  |  |  | 0.31 | -0.20 | 0.63 |
| *H_2_* |  |  |  |  |  |  |  |  |  |  |  |  |  |  |  |  | 0.04 | 0.56 |
| *CH_4_* |  |  |  |  |  |  |  |  |  |  |  |  |  |  |  |  |  | 0.34 |

**Table S3** Correlation coefficients and R^2^ values for the relation of outlet-conductivity to sCOD and SCCA (Figure 4)

| Feedstock | sCOD over Conductivity (outlet) | | SCCA over conductivity (outlet) | |
| --- | --- | --- | --- | --- |
|  | Pearson R | R^2^ | Pearson R | R^2^ |
| **MZ** | 0.833 | 0.684 | 0.805 | 0.637 |
| **30 % straw** | 0.741^a^ | 0.542^a^ | 0.690 | 0.452 |
| **66 % straw** | 0.742 / 0.687^a^ | 0.540 / 0.460^a^ | 0.728 | 0.520 |

^a^ without lactic-acid fermentation
